# The Biogenesis, Biology, and Clinical Significance of Exosomal PD-L1 in Cancer

**DOI:** 10.3389/fimmu.2020.00604

**Published:** 2020-04-07

**Authors:** Yanyan Tang, Ping Zhang, Yumin Wang, Jinpeng Wang, Min Su, Ying Wang, Lianqing Zhou, Jumei Zhou, Wei Xiong, Zhaoyang Zeng, Yujuan Zhou, Shaolin Nie, Qianjin Liao

**Affiliations:** ^1^Hunan Cancer Hospital and the Affiliated Cancer Hospital of Xiangya School of Medicine, Central South University, Changsha, China; ^2^Department of Colorectal Surgery, Hunan Cancer Hospital and the Affiliated Cancer Hospital of Xiangya School of Medicine, Central South University, Changsha, China; ^3^Central Laboratory, The Affiliated Cancer Hospital of Xiangya Medical School, Central South University, Changsha, China; ^4^College of Computer and Information Engineering, Hunan University of Technology and Business, Changsha, China; ^5^The Key Laboratory of Carcinogenesis and Cancer Invasion of the Chinese Ministry of Education, Cancer Research Institute, Central South University, Changsha, China; ^6^Urban Vocational College of Sichuan, Chengdu, China; ^7^Department of Radiotherapy, Hunan Cancer Hospital and the Affiliated Cancer Hospital of Xiangya School of Medicine, Central South University, Changsha, China

**Keywords:** exosomal PD-L1, biogenesis, immunosuppression, T cells, biomarker, immunotherapy

## Abstract

The exosome serves as a trafficking vehicle for transport of programmed death-ligand 1 (PD-L1) into receptor cells. In tumor microenvironment, distant tumor cells can remotely attack activated T cells by exosomal PD-L1. Here, we summerize the biogenesis and transport process of exosomal PD-L1. Then, we focus on the cancer biology of exosomal PD-L1 in immunosuppression and the mechanism by which it inhibits T cells. Finally, we highlight the prospects of exosomal PD-L1 as a tumor biomarker and its significance in immunotherapy. In addition, we discuss the new challenges faced in researching and utilizing exosomal PD-L1. This review may shed light on the exosomal PD-L1 from the bench to the clinic. Exosomes serve as trafficking vehicles for transport of programmed death-ligand 1 (PD-L1) into receptor cells. In tumor microenvironment, distant tumor cells can remotely attack activated T cells through exosomal PD-L1. Here, we have summarized the biogenesis and transport of exosomal PD-L1. Next, we focused on the cancer biology of exosomal PD-L1 in immunosuppression and the mechanism by which it inhibits T cells. Finally, we highlighted the prospects of exosomal PD-L1 as a tumor biomarker and its significance in immunotherapy. In addition, we have discussed the new challenges faced in studying and utilizing exosomal PD-L1. This review may shed light on the translation of exosomal PD-L1 from bench to clinic.

## Introduction

Programmed death-ligand 1 (PD-L1, also known as CD274) is a membrane-bound ligand found on the cell surface of many tumor cell types that are upregulated in oncogenic lesions (1). PD-L1 binds programmed cell death protein-1 (PD-1, also known as CD279) on T cells, suppressing their activation (2). Tumor cells can co-opt this pathway to induce immune evasion (3, 4).

In addition to cell surface, PD-L1 is also found on the surface of exosomes, where it is called exosomal PD-L1. Exosomes are extracellular vesicles (EVs) with sizes ranging from 30 to 150 nm (5–7). Chen et al. have recently shown that PD-L1 is preferentially enriched in exosomes, when compared with that in melanoma microvesicles, suggesting that exosomes are the primary source of PD-L1 among EVs in this cancer type (8). Other studies have confirmed that exosomal PD-L1, EVs containing PD-L1, and exosomal PD-L1 mRNA can be detected in melanoma, prostate cancer, breast cancer, glioblastoma, head and neck cancer, lung cancer, and other tumors (8–15) (Table 1).

**Table 1:** Examples of exosomal PD-L1 in tumor immune evasion.

**Cancer type**	**Role of exosomal PD-L1 in cancer**	**Potential mechanism**	**References**
Metastatic melanoma	Exosomal PD-L1 facilitates the progression of melanoma *in vitro* and *in vivo*, and stratifies clinical responders to pembrolizumab and non-responders	Suppresses the function of CD8^+^ T cells to mediate immunosuppression	(8)
PC, CRC	Exosomal PD-L1 promotes tumor growth across different cancer types through suppression of the immune system	Suppresses T cell activity in the draining lymph node, and suppresses systemic anti-tumor immunity	(13)
Breast cancer	Exosomal PD-L1 suppresses killing of breast cancer cells by T cells and promote tumor growth	Inhibits CD3/CD28-induced ERK phosphorylation and NF-κB-mediated activation of T cells to attenuate anti-tumor immunity and suppresses T cell activity	(9)
Glioblastoma	EVs containing PD-L1 DNA could provide a surrogate marker of tumor volume and possibly help in real-time monitoring of cancer progression	Blocks TCR-mediated T cell activation to mediate immune evasion	(10)
HNSCC	The level of exosomal PD-L1 correlates with disease activity in patients with HNSCC, UICC stage, and lymph node status	Downregulates expression CD69 in effector T cells to suppress T cell-mediated killing	(11)
NSCLC	Exosomal PD-L1 promotes tumor growth *in vitro* and *in vivo*	Exosomal PD-L1 induces apoptosis and inhibits IFN-γ production in CD8+ T cells, and inhibits T cell activity	(15)
GC	Exosomal PD-L1 is associated with poor prognosis of patients with GC	Decreases expression of CD69 and PD-1 on T cell surface	(14)

*PC, prostate cancer; EVs, extracellular vehicles; CRC, colorectal cancer; HNSCC, head and neck squamous cell carcinoma; UICC, Union for International Cancer Control; NSCLC, non-small cell lung cancer; GC, gastric cancer*.

Tumor-derived exosomal PD-L1 combines the advantages of exosomes and PD-L1 to mediate an immunosuppressive effect. Exosomal PD-L1 is more stable and not easily degraded by proteolytic enzymes (14). Additionally, exosomal PD-L1 might exert stronger immunomodulatory effects in circulation and tumor microenvironments. Therefore, the clinical value of exosomal PD-L1 is more significant. However, the origin of exosomal PD-L1, its intracellular transportation, and its interactions with other cells after being secreted are yet to be fully elucidated.

In this review, we have focused on the biogenesis, roles, and mechanisms of exosomal PD-L1. Moreover, we have discussed the potential application of exosomal PD-L1 as a novel diagnostic biomarker and a new target for immunotherapy.

## Biogenesis of Exosomes and Exosomal PD-L1

### Where Does Exosomal PD-L1 Come From?

**Figure 1:**
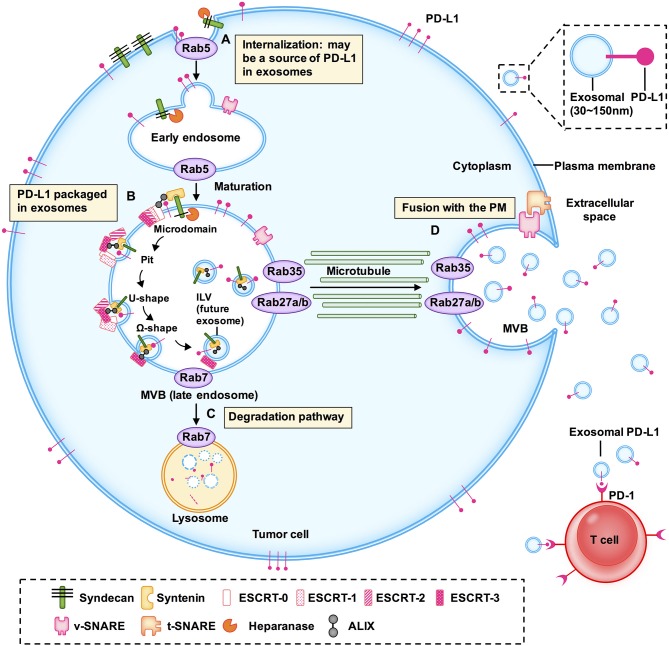
Models of the potential biogenesis and transport mechanisms of exosomal PD-L1. Exosomal PD-L1 may arise from endocytosis of the plasma membrane (PM). **(A)** Early endosomes are membrane-bound vesicles formed by the internalization of the inner PM. **(B)** The membrane of early endosomes bulges inward to form intraluminal vesicles (ILV) within the lumen of multivesicular bodies (MVBs). **(C)** When MVBs fuse with lysosomes, their cargo, including ILVs, is degraded. **(D)** When MVBs fuse with the plasma membrane to release ILVs containing PD-L1, this PD-L1 is called exosomal PD-L1. This is the final and key step of exosome secretion. Endosomal sorting complexes (ESCRT; e.g., ESCRT-0, ESCRT-1, ESCRT-2, ESCRT-3), RAS-related protein GTPases (Rabs; e.g., Rab5, Rab7, RAB27A/B, RAB35), ALG-2 interacting protein X (ALIX), soluble N-ethylmaleimide sensitive factor attachment protein receptors (SNAREs) and other key enzymes regulate the transport and sorting processes of exosomes.

Given that exosomes have a bilayer membrane structure of endosomal origin and PD-L1 is a transmembrane protein, exosomal PD-L1 may arise from endocytosis of the plasma membrane (PM). Poggio et al. confirmed that exosomal PD-L1 does not appear to originate directly from the endoplasmic reticulum (ER) or Golgi but rather from the surface in PC3 prostate cancer cells (13). As reviewed elsewhere by Colombo et al., endocytosis of the PM forms early endosomes (16). Previous studies indicated that PD-L1 internalized through the PM might be a source of PD-L1 in exosomes (Figure 1A).

RAS related proteins (Rabs) belong to the Ras superfamily of small GTPases. Accumulating evidence has shown that Rab family members, including Rab5, Rab7, Rab27a, Rab27b, and Rab35, play a vital role in the biogenesis, transport, and secretion of exosomes (17–20). Rab5 localizes on early endosomes and the PM, mediates endocytosis, and generates and maintains the early endosomes (21, 22). Heparanase is a mammalian endoglycosidase that cleaves heparan sulfate proteoglycans into heparan sulfate at the cell surface. It assists in driving exosome secretion, alters exosome composition, and facilitates biogenesis of exosomes, thereby promoting tumor progression (23). Heparanase acts through syntenin-1, ALG2-interacting protein X (ALIX), and syntenin-ALIX complex to stimulate intraluminal budding into multivesicular bodies (MVBs), leading to exosome biogenesis (24). However, the precise mechanism of formation of exosomal PD-L1 has not been fully elucidated and it may vary among different cell types. The pivotal questions that should be resolved in the immediate future concern the localization of PD-L1 in cells: how much of it is in the PM, how much of it is on the exosomes, and the underlying regulatory mechanism. Therefore, it is important to further investigate the molecules regulating the biogenesis and distribution of exosomal PD-L1.

### PD-L1 Packaged in Exosome at Subcellular Structure

During the maturation of early endosomes to late endosomes, inward budding of the early endosomal membrane forms intraluminal vesicles (ILVs) (5, 16). A late endosome containing several ILVs is called a multivesicular body (MVB) (25). Poggio et al. have shown that PD-L1 and the exosomal marker hepatocyte growth factor receptor substrate (HRS) co-localize with CD63, indicating that PD-L1 is packaged in exosomes and the pre-exosomal PD-L1 is localized in the subcellular structure (13) (Figure 1B).

Exosomes are produced as ILVs in the lumen of endosomes, which involves a specific sorting mechanism. Endosomal sorting complexes required for transport (ESCRT) complexes play a driving role in membrane formation and rupture in a certain order. Following are the ESCRT complexes: ESCRT-0, ESCRT-1, ESCRT-2, and ESCRT-3 (26). ESCRT-0 recognizes and binds ubiquitinated proteins on the cargo and sorts the cargo into spatially restricted areas on the endosomal membrane. Cargo sorting is supported by clathrin (27–29). Studies have demonstrated that PD-L1 colocalizes with HRS (the component of ESCRT-0) and CD63 in MVBs (8, 9). ESCRT-1, which contains tumor susceptibility gene 101 protein (TSG101), and ESCRT-2 regulate the initial deformation of membrane into buds with sequestered cargo and may be involved in cargo transfer (29). ESCRT-3 together with the vacuolar protein sorting 4 homolog (VPS4) drives membrane constriction and scission of ILVs (29).

ESCRT-associated ALIX and syntenin-ALIX complex together stimulate the intraluminal budding (24). In addition, ALIX controls exosomal cargo incorporation. PD-L1 is mis-secreted in the absence of ALIX (24, 30). ALIX can also regulate PD-L1 surface presentation in basal-like breast cancer (BLBC) cells (30, 31). ALIX depletion prevents the incorporation of PD-L1 into exosomes and increases PD-L1 expression on the cell surface (30).

Baietti et al. showed that the syndecan-syntenin-ALIX complex originates from endosomal membranes and from the fusion of MVBs with the cell surface, bypassing lysosomes (32). Further, Roucourt et al. reported that heparanase activates the syndecan-syntenin-ALIX exosome biogenesis pathway. Therefore, targeting these regulatory proteins may inhibit the intracellular biogenesis of exosomes and packaging of exosomal PD-L1.

### The Degradation and Secretion of Exosome

When MVBs fuse with lysosomes, their cargo, including ILVs, is degraded (16, 18, 33). When MVBs are transported along microtubules and fuse with the PM, the ILVs are released into the extracellular environment as exosomes; this is the final and key step in exosome secretion (34) (Figures 1C,D). ISGylation is an ubiquitin-like modification that controls exosome release by targeting the ESCRT complex. Beltri et al. reported that IFN-I induces expression and conjugation of ISG15 to TSG101, a component of ESCRT-1. ISGylation of TSG101 promotes fusion of MVBs with lysosomes and their degradation, thus inhibiting exosome secretion (35). ESCRT silencing regulates the formation of MVBs but does not result in their complete elimination, which indicates the presence of independent mechanisms.

Rab7, which is located in the MVBs, mediates maturation of MVBs and their trafficking to lysosomes (21). Rab27a/b regulates MVB-exosome secretion (36). Although Rab27a is mainly located on CD63-positive MVBs, it is predominantly present in the trans-Golgi network (TGN). Rab27a and Rab27b share some, but not all, roles, and most likely function through synaptotagmin-like 4 (Slp4, also known as SYTL4) and exophilin 5 (Slac2b, also known as EXPH5) effector proteins, respectively, to elicit exosome secretion by promoting MVB distribution to the cell periphery (25). Vacuolar protein sorting protein 33B (VSP33B) may regulate exosome maturation and release by interacting with guanosine diphosphate dissociation inhibitor 2 (DGI2), Rab11a, and Rab27a. However, details of how these regulators fine-tune exosome maturation and secretion require further elucidation (37). Knockdown of Rab27a significantly inhibits exosome secretion and growth of 4T1 mouse mammary tumor cells, and strikingly augments anti-PD-L1 therapeutic efficacy (9). Rab27b facilitates exosome shedding in acute myeloid leukemia (AML) KG-1a cells (20). Rab35 controls MVB transport and impacts the docking process (34). It also transports podocalyxin (a sialomucin associated with cancer aggressiveness) to the PM (38).

Soluble N-ethylmaleimide-sensitive fusion factor attachment protein receptors (SNAREs) have been recognized as key components of complexes that drive membrane fusion (39). SNAREs contribute to the release of ILVs as exosomes. V-SNARE on the MVB membrane binds to t-SNAREs on the PM, forming a stable SNARE complex, which mediates the fusion of MVB with the PM (34, 39). nSMase2 is a rate-limiting enzyme of ceramide biosynthesis and triggers secretion of exosomes via a ceramide-dependent pathway (40). Ceramide triggers budding of exosomes into MVBs (41). GW4869, a nSMase inhibitor, reduces secretion of exosomes (13).

Here, we discussed the biogenesis, transport, degradation, and secretion of exosomes and their regulatory processes, as well as the localization of exosomal PD-L1 in subcellular structure (Figure 1). We have summarized the proteins that are involved in the biogenesis, transport, sorting, and secretion of exosomes in Table 2, including ESCRT, ESCRT-associated protein, Rabs, SNAREs, and other enzymes (17, 24, 26, 43, 44). These studies suggested that targeting any step of the biogenesis or a regulatory molecule of exosomes might affect the level of exosomal PD-L1, and may result in a new method for inhibition of tumor metastasis and a novel strategy for addressing the resistance of tumors to immunotherapy.

**Table 2:** Composition and function of complexes and key enzymes related to exosome biogenesis, sorting, transport and secretion.

**Complexes**	**Subunits**	**Localization**	**Function**	**References**
ESCRT	ESCRT-0 (HRS, STAM)	MVBs	Recognizes and binds ubiquitinated proteins and sorts them into spatially restricted areas on the endosomal membrane; HRS recognizes the mono—ubiquitinated proteins and recruits TSG101	(26, 29, 42)
ESCRT	ESCRT-1 (TSG101, VPS28, VPS37, MVB12), ESCRT2 (VPS36, VPS22, VPS25)	MVBs	Regulates the initial deformation of membrane into buds with sequestered cargo and may be involved in cargo transfer	(26, 29, 42)
ESCRT	ESCRT-3 (VPS2, VPS20)	ILV, MVBs	Drives membrane invagination and separation, and subsequent vesicle scission	(26, 29, 42)
AAA ATPases	VPS4	ILV	Interact with ESCRT-3 to cause constriction and scission of ILV	(29)
ESCRT-associated protein	ALIX	ILVs, MVBs	Controls exosomal cargo incorporation and regulates sorting of PD-L1 onto ILVs; ALIX and syntenin-ALIX complex stimulate intraluminal budding	(24, 30)
Rabs	Rab5	Early endosomes, PM	Mediates endocytosis and generation and maintenance of early endosomes	(21, 22)
Rabs	Rab7	MVBs	Mediates maturation and trafficking of MVBs to lysosomes	(21)
Rabs	Rab27a	MVBs	Involved in the fusion of MVBs to the PM	(13, 25)
Rabs	Rab27b	MVBs	Promotes formation and stability of MVB docking and facilitates exosome shedding	(20, 25)
Rabs	Rab35	MVBs	Controls MVB transport and influences the docking process	(34)
SNAREs	v-SNARE, t-SNARE,	Widespread distribution in the endosomal system	Drive membrane fusion and mediate fusion of MVBs with the PM	(34, 39)
Enzyme	Heparanase	Endosome membrane, PM	Exogenous heparanase impacts intraluminal budding and, therefore, exosome biogenesis	(24)
Enzyme	nSMase2/SMPD3	PM, endosomes	Regulates biosynthesis of ceramide and promotes budding of intravesicular vesicles	(13, 40)

*ESCRT, endosomal sorting complexes required for transport; HRS, hepatocyte growth factor receptor substrate; TSG101, tumor susceptibility gene 101 protein; STAM, signal transducing adaptor molecule; MVB, multivesicular bodies; PM, plasma membrane; VPS, Vacuolar protein sorting homolog; ALIX, ALG2-interacting protein X; Rabs, RAS related proteins; TGN, trans Golgi network; SNARE, soluble N-ethylmaleimide-sensitive fusion factor attachment protein receptor; nSMase2, neutral sphingomyelinase 2; SMPD3, Sphingomyelin Phosphodiesterase 3*.

However, mechanisms regulating the biogenesis and transport of exosomes and exosomal PD-L1 are only partially understood. In addition, there are no reports on whether the regulators of the lysosomal degradation pathway can decrease the level of exosomal PD-L1. This problem deserves further study.

## Cancer Biology of Exosomal PD-L1

### Exosomal PD-L1 Induces Immunosuppressive Microenvironments

**Figure 2:**
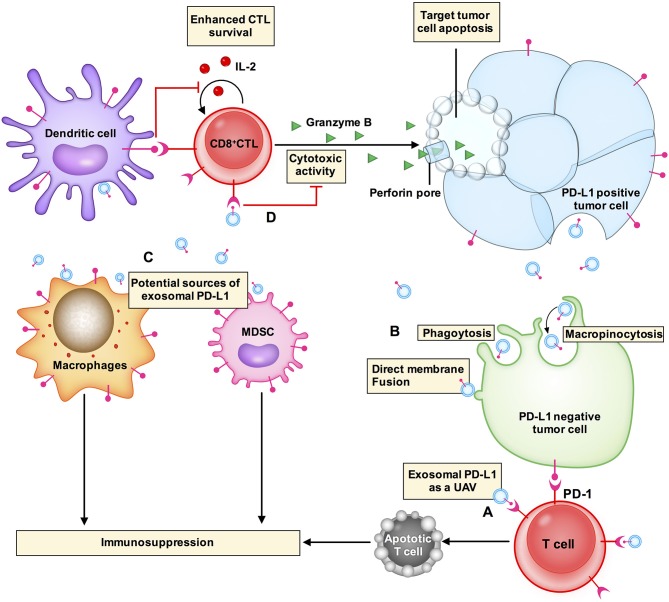
Exosomal PD-L1 induces immunosuppressive microenvironments. **(A)** Exosomal PD-L1 (small blue circle with a red stick) can fight circulating T cells (cell in red) remotely, even before T cells approach tumor cells, like a biological unmanned aerial vehicle (UAV). **(D)** CD8^+^ CTLs target and induce apoptosis of tumor cells through cytotoxic activity. Exosomal PD-L1 can block cytotoxicity in CD8^+^ T cells. **(B)** Exosomal PD-L1 can be transferred to a variety of cell types, including PD-L1-negative tumor cells (cell in green), macrophages and dendritic cells in the tumor microenvironment. The potential mechanisms of receptor cell uptake of exosomal PD-L1 include phagocytosis, macropinocytosis, or direct fusion of exosomes with the plasma membrane. **(C)** PD-L1 is not only expressed in tumor cells but is also expressed in other immune cells, e.g., macrophages, dendritic cells, and myeloid-derived suppressor cells (MDSCs). PD-L1 expression from these other cells plays a role in glioblastoma immunosuppression. These other cells may also be sources of exosomal PD-L1.

Exosomal PD-L1 secreted by melanoma, breast cancer, and gastric cancer cells has immunosuppressive effects (8, 9, 14). Chen et al. proposed a model of melanoma in which exosomal PD-L1 can fight circulating T cells remotely, even before T cells approach the melanoma cells, like a biological unmanned aerial vehicle (UAV) (8) (Figure 2A). Poggio et al. reported that exosomal PD-L1 systematically acts to suppress the anti-tumor immune response in prostate cancer, and its genetic blockage promotes T cell activity in draining lymph nodes to induce systemic anti-tumor immunity and memory (13). Fan et al. showed that exosomal PD-L1 levels reflect immunosuppression in patients with metastatic gastric cancer (GC). In addition, exosomal PD-L1 levels were negatively correlated with granzyme B (GzmB) levels (14) (Figure 2D).

Yang et al. have demonstrated that exosomes transport PD-L1 from PD-L1-positive to PD-L1-negative breast cancer cells (9). Using flow cytometry, it has been revealed that PD-L1 transported by exosomes is located on the surface of target cells and could bind to PD-1 (9). These results suggest that exosomes can transfer functional PD-L1 to other cells that have little or no expression of PD-L1 and can play an important role in immunosuppression. There are two common mechanisms by which receptor cells uptake exosomes: (i) endocytosis or internalization, which is the most common mechanism of uptake of exosomes by recipient cells and includes phagocytosis or macropinocytosis (16, 45) and (ii) binding of exosomes to the recipient cell surface, which leads to direct fusion of the exosomes with the PM (45–47) (Figure 2B). Nevertheless, the mechanism by which exosomal PD-L1 transmits PD-L1 to target cells remains poorly understood.

In the tumor microenvironment, PD-L1 is expressed not only in tumor cells but also in other cell types including macrophages, dendritic cells (DCs), and myeloid-derived suppressor cells (MDSCs) (48). PD-L1 expressed in these cells plays roles in glioblastoma immunosuppression (10, 48). These cell types may also be sources of exosomal PD-L1 (Figure 2C). Additionally, exosomal PD-L1 can be transferred to a variety of cell types *in vitro*, including tumor cells, human monocyte cell line THP1, macrophages, and DCs (9). *In vivo*, immunofluorescent staining has revealed that exosomal PD-L1 induces PD-L1-positive tumor-infiltrating macrophages; however, the staining was negative in DCs (9). This could be explained by the presence of fewer DCs or some unknown mechanisms in the tumor microenvironment that require further elucidation. Additional questions that need to be addressed include how macrophages fail to degrade exosomal PD-L1 and whether this affects the therapeutic efficacy of anti-PD-1 and anti-PD-L1 antibody treatments.

Overall, the actions of exosomal PD-L1 seem to both directly regulate T cells and indirectly modulate other immune cells to make the tumor microenvironment immune-suppressive and avoid anti-tumor immune response.

### Exosomal PD-L1 Blocks T Cell Activation

CD8^+^ cytotoxic T lymphocytes (CTLs) target and induce tumor cell apoptosis through cytotoxic activity. Exosomal PD-L1 blocks the cytotoxicity of CD8^+^ T cells. Exosomes with high levels of PD-L1, COX-2, CTLA-4, CD15s, or CD44v3, can induce apoptosis in activated CD8^+^ T cells (49). Exosomal PD-L1 inhibits CD8^+^ T cells and facilitates the progression of melanoma *in vitro* and *in vivo* (8). Poggio et al. reported that exosomal PD-L1 suppresses T cell activation *in vitro* and suppresses T cell activity in draining lymph nodes in prostate cancer (13). In addition, exosomal PD-L1 from other cancer cell lines, such as colon (RKO) and lung (HCC827), has similar functions in blocking T cell activation (9).

**Figure 3:**
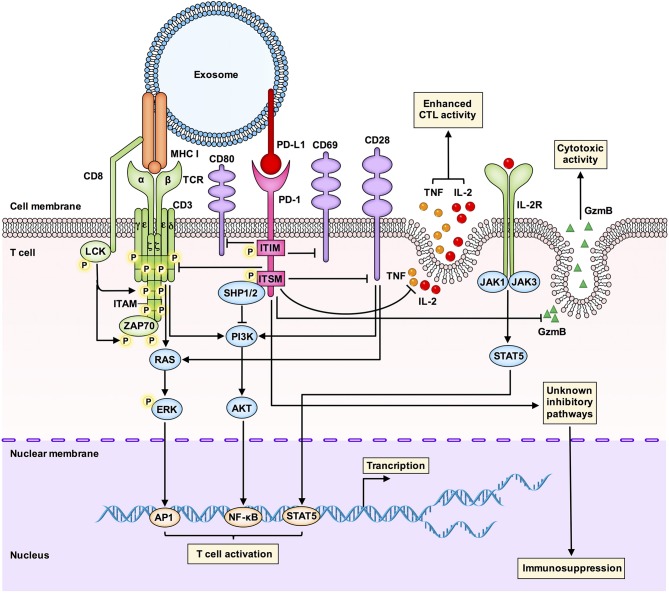
An integrated overview of the immunosuppressive signaling pathways involved in the interaction between exosomal PD-L1 and T cells. Tumor-derived exosomal PD-L1 can bind to PD-1 on T cells to inhibit T cell activation and cytotoxicity. Interactions between PD-L1 and PD-1 can induce phosphorylation of the cytoplasmic immunoreceptor tyrosine based inhibitory motif (ITIM) and the immunoreceptor tyrosine based switch motif (ITSM). The phosphorylated ITIM and ITSM recruit the SHP-1 and SHP-2 protein tyrosine phosphatases to attenuate activating signals of T cells. Exosomal PD-L1 blocks TCR-mediated T cell activation. Exosomal PD-L1 can deliver inhibitory signals to activate T cells by downregulating CD69, CD28, and CD80 expression on the surface of T cells. Exosomal PD-L1 inhibits CD3/CD28-triggered T cell activation signaling pathways, including CD3/CD28-induced ERK phosphorylation and NF-κB activation. Exosomal PD-L1 inhibits the proliferation, cytokine production and cytotoxicity of CD8^+^ T cells by inhibiting the expression of granzyme B (GzmB) and inhibiting the production of interleukin-2 (IL-2) and tumor necrosis factor (TNF). GzmB is the effector molecule of cytotoxic activity in T cells, and IL-2 and TNF can enhance cytotoxic T lymphocyte (CTL) activation and survival via the JAK1/JAK3-STAT5 pathway. Exosomal PD-L1 may also affect immunosuppression through other unknown mechanisms, which are worth further study.

How does exosomal PD-L1 inhibit the functions of T cells? As shown in Figure 3, several pathways are involved in the action of exosomal PD-L1.

First, glioblastoma-derived EVs containing PD-L1 block T cell receptor (TCR)-mediated T cell activation (10). During T cell maturation, any defect in the TCR-CD3 complex may result in T cell dysfunction and immunosuppressive clinical symptoms. The CD3-ζ chain in T cells promotes the signal transduction of the TCR-CD3 complex (50, 51), and inhibition of the CD3-ζ chain in T cells affects the function of T cells (52).

Second, Theodoraki et al. have shown that exosomal PD-L1 suppresses the functions of CD8^+^ effector T cells. The levels of PD-L1 in circulating exosomes were positively correlated with the ability of exosomal PD-L1 to inhibit the activation of CD8^+^ effector T cells. Further, involvement of the PD-L1-mediated pathway in the downregulation of surface expression of CD69 (a T cell activation marker) in activated T cells after coincubation with exosomal PD-L1 has also been confirmed (11).

Third, exosomal PD-L1 inhibits CD3/CD28-triggered T cell activation signaling pathways. Researchers have found that exosomal PD-L1 significantly inhibits CD3/CD28-induced ERK phosphorylation and NF-κB activation (9).

Fourth, melanoma cell-derived exosomal PD-L1 inhibits cytokine production and cytotoxicity of CD8^+^ T cells by decreasing the expression of GzmB and inhibiting the production of interleukin-2 (IL-2) and tumor necrosis factor (TNF) (8). IL-2 can enhance CTL activation and survival via the Janus kinase 1 (JAK1)/JAK3-STAT5 pathway. Another study has also shown that exosomal PD-L1 promotes tumor growth *in vivo* by inhibiting the secretion of GzmB from T cells (9).

### Regulation of Exosomal PD-L1 on T Cells

During tumor progression, PD-L1 is expressed on the surface of tumor cells and tumor infiltrating lymphocytes (TILs). Interferon-γ (IFN-γ) signaling results in expression of PD-L1 on the surface of various tumor cells as well as on the exosomal membrane (8, 53, 54). In melanoma cells, the IFN-γ-JAK1/JAK2-STAT1-IRF1 axis regulates PD-L1 expression as melanoma cells can mediate adaptive resistance by upregulating PD-L1 expression in response to IFN-γ released by CTLs (55).

The level of exosomal PD-L1 secreted by melanoma cells is markedly increased upon IFN-γ treatment, and exosomal PD-L1 primarily targets PD-1^+^ CD8^+^ T cells (8). The level of circulating exosomal PD-L1 was positively correlated with the level of circulating IFN-γ, both of which have been shown to be indicative of poor prognosis (8, 56). Similar to PD-L1 found on the cell-surface membrane and melanoma-derived exosomal PD-L1, the level of prostate cancer-derived exosomal PD-L1 is increased in response to IFN-γ (13). Ricklef et al. have shown that even EVs containing a low level of PD-L1 have the potential to inhibit T cell activation when PD-L1 is upregulated by IFN-γ (10). Additionally, a recent study reported the regulation from another perspective, in which exosomal PD-L1 inhibited IFN-γ secretion by Jurkat T cells (15). The above studies indicated that IFN-γ is a double-edged sword for immune system, the regulation between IFN-γ and exosomal PD-L1 is different under various situations. While on one hand, TIL-derived IFN-γ upregulates exosomal PD-L1, thus protecting tumor cells from the surveillance of the immune system; on the other hand, CD8^+^ T cells inhibit the proliferation of tumor cells by secreting IFN-γ and exosomal PD-L1 may reduce the level of CD8^+^ T cells-derived IFN-γ through PD-L1/PD-1 interaction.

Nevertheless, IFN-γ did not increase the number of exosomes secreted, and there was no direct evidence of how IFN-γ affects the production of exosomal PD-L1. This problem needs to be further elucidated.

Based on results of current studies, we have illustrated the pathways and mechanisms of exosomal PD-L1 mediated immunosuppression. The role of exosomal PD-L1 associated with poor prognosis of patients may be the cause of low response to immunotherapy. Targeting exosomal PD-L1 regulatory molecules might inhibit the function of exosomal PD-L1, which may improve the efficacy of immunotherapy.

## Clinical Significance of Exosomal PD-L1 in Cancer

### Exosomal PD-L1 As a Potential Biomarker for Efficacy of Immunotherapy

Based on the knowledge that immunotherapy is expensive and toxic, it is important to know which patients will respond to it. At present, clinical treatment is in urgent need of biomarkers that can accurately predict the efficacy of immunotherapy to optimize the selection of therapies for patients with tumor so that the patients achieve individual treatment and precision medicine.

It is generally believed that overexpression of PD-L1 can improve the response rate of immunotherapy. In the Keynote 052 study, patients with urothelial cancer received pembrolizumab. The expression of PD-L1 ranged from 1 to 10%, the therapeutic effect of drugs became better with increase in PD-L1 expression. The objective response rate (ORR) of patients with PD-L1 expression higher than 10% was significantly higher than that of patients with PD-L1 expression lower than 1% (39 vs. 11%) (57).

However, the results of CheckMate032 seem paradoxical in this conclusion. The results showed that there was no significant difference in the efficacy of nivolumab between patients with high expression of PD-L1 (>1%) and those with low expression of PD-L1 (<1%) (24 vs. 26.2%) (58).

There are possible explanations for the above seemingly mutually exclusive results. (i) The cut-off values and scoring criteria used in different clinical trials are inconsistent, and the various IHC antibodies and test platforms used in each experiment will lead to different results. (ii) The upregulation of PD-L1 can be caused by many reasons. Different PD-L1 regulatory mechanisms may influence immunotherapy. (iii) The expression of PD-L1 is heterogeneous, including inter-tumor heterogeneity and intra-tumor heterogeneity. The heterogeneity may lead to inconsistent expression of PD-L1 in biopsy tissues and surgically resected tissues (59). With the progress of treatment and advancement of disease, PD-L1 expression will change correspondingly. Kelly et al. found that in 50% of patients with advanced esophageal adenocarcinoma who received radiotherapy and chemotherapy, negative PD-L1 expression changed to positive expression (60). Therefore, if PD-L1 is used as a biomarker for predicting efficacy, it is necessary to unify the criteria and consider various influencing factors.

Exosomal PD-L1 is better as a biomarker in some aspects. Compared with tumor cell PD-L1, exosomal PD-L1 can be widely spread and more easily attached to target cells, which can promote immune escape more effectively. It reflects the state of the whole body system, and can predict the dynamic progress of the disease. The methods for detecting exosomal PD-L1 include ELISA, flow cytometry, and ddPCR. The methods are non-invasive and can be performed at multiple time points, which could overcome the problems related to tumor heterogeneity. Use of exosomal PD-L1 as a biomarker combines the advantages of exosomes and PD-L1.

The level of circulating exosomal PD-L1 in patients with metastatic melanoma is significantly higher than the level in healthy persons (8). Exosomal PD-L1 can be adopted as an important blood biomarker due to the complexity of exosome cargos and its detectability in blood. A study of a small sample of population found that the expression of PD-L1 mRNA in plasma-derived exosomes of patients with melanoma and NSCLC was correlated with the efficacy of anti-PD-1 immunotherapy (12). The level of PD-L1 mRNA in exosomes is significantly decreased in plasma of patients who were treated with PD-1 therapy (12). When the level of PD-L1 mRNA in exosomes increases, it may transmit immunosuppressive signals to T cells. At this time, the efficacy of anti-PD-1 therapy is poor, and patients develop disease progression (12). This indicates that it is feasible to detect dynamic changes in PD-L1 mRNA expression in exosomes, which may provide relevant information on clinical outcome to anti-PD-1 therapy. Overexpression of exosomal PD-L1 is positively correlated with clinicopathological features of tumors, including stage, lymph node metastasis, and disease progression (11). For instance, the level of exosomal PD-L1 can be used to distinguish patients with metastatic melanoma from healthy persons (8). Significant elevation in the level of exosomal PD-L1 was observed in patients with active disease (AD) compared with those patients who had no evidence of disease (NED) in head and neck cancer. In other words, patients with a high level of PD-L1-positive exosomes had faster disease progression than patients with a low level of PD-L1-positive exosomes in the plasma (11). Additionally, a higher level of PD-L1 in circulating exosomes was associated with a stronger ability to inhibit CD8^+^ effector T cells (11). There was also a positive correlation between PD-L1 expression and EVs containing PD-L1 DNA in patients with glioblastoma. These data suggest that exosomal PD-L1 or EVs containing PD-L1 DNA have potential as clinical biomarkers and have prognostic value. We have summarized the quantitation of exosomal PD-L1 obtained from patients with different tumor types and clinical effects of the exosomal PD-L1 on PD-1 therapy (Table 3).

**Table 3:** Quantitation of exosomal PD-L1 and its clinical effects on PD-1 therapy.

**Cancer type**	**How the studies were done**	**Quantitation of exosomal PD-L1 in patients**	**Clinical effects on PD-1 therapy**	**Clinical implications and significance**	**References**
Metastatic melanoma	PD-L1 was high in exosomes from metastatic melanoma by WB. Exosome PD-L1 could bind to PD-1 on T cells by electron microscopy, ELISA and confocal microscopy analysis. To predict the efficacy of immunotherapy by detecting the pre-treatment level of PD-L1	0.1~5.4 ng/ml	Exosomal PD-L1 >2.43 was associated with a better response to anti-PD-1 therapy on ORR, PFS and OS	For providing a rationale for application of exosomal PD-L1 as a predictor for anti-PD-1 therapy in melanoma	(8)
HNSCC	Exosomes isolated by size exclusion chromatography were captured on CD63 beads. The correlation of percentages and mean fluorescence intensities of PD-L1^+^ exosome with the patients' clinicopathological data by FCM analysis	Approximately 25~83% of exosomal PD-L1/ml plasma in AD patients; 15~35% of exosomal PD-L1/ml plasma in NED patients	NA	To predict HNSCC patients' disease activity, the UICC stage and the lymph node status by levels of exosomal PD-L1	(11)
Melanoma	To investigate response of exosomal PD-L1 to nivolumab and pembrolizumab in patients with melanoma and NSCLC, blood was obtained at time point 0 and after 2 months	140~2,500 (copies/ml) 95~190 (copies/ml) 0~90 (copies/ml)	CR+PR SD PD	PD-L1 mRNA in exosomes is associated with response to anti-PD-1 in melanoma and NSCLC	(12)
NSCLC		330~1,700 (copies/ml) 380~530 (copies/ml) 0~650 (copies/ml)	PR SD PD		(12)

*ORR, objective response rate; FCM, flow cytometry; PFS, progression free survival; OS, overall survival; HNSCC, head and neck squamous cell carcinoma; AD, active disease; NED, no evidence of disease; NA, not applicable; CR, complete response; PR, partial response; SD, stable disease; PD, progression of disease*.

### Implications of Exosomal PD-L1 for Challenges in Immunotherapy

There are evidences that PD-1/PD-L1 is a viable target for cancer immunotherapy (61, 62). PD-1 or PD-L1 immune checkpoint inhibitor antibodies can reverse PD-L1-mediated immunosuppression (63). The advent of immune checkpoint inhibitors has become a promising anti-cancer strategy for many tumor types (64, 65). For instance, the efficacy and survival outcomes of patients treated with nivolumab are better than those of patients treated with conventional chemotherapy (66, 67).

However, like many other anti-cancer therapies, immunotherapy also faces great challenges. While anti-PD-1 therapy is strikingly effective in some patients, others patients do not respond to it. For example, pembrolizumab administration was associated with an overall ORR of 33% in patients with advanced melanoma (clinicaltrials.gov identifier: NCT01295827) (68). In other words, the response rate of most patients with melanoma was low in this clinical trial.

The reasons for the low ORR of immunotherapy may be as follows. First, there may be other molecules or mechanisms mediating immunosuppression in tumors. In this case, single anti-PD-1 therapy would not be enough to augment therapeutic efficacy. The combination of anti-PD-1 therapy and other immunotherapy may enhance the killing effect of tumor cells. For example, the ORR was 58% for the nivolumab-plus-ipilimumab group in CheckMate 067 (65, 69). In addition, radiotherapy affects the expression of circulating PD-L1. The significant increase in the level of soluble PD-L1 in patients with hepatocarcinoma after radiotherapy suggests that the combination of radiotherapy and circulating PD-L1 inhibitors may be more effective (70).

Second, PD-1 inhibitors revealed signs of immunotherapy resistance and did not show long-lasting response, greatly reducing the efficacy of treatment or resulting in direct failure. With the rapid development of immunotherapy, this problem may become more serious in the future.

The existing immunotherapy can only target and block PD-1/PD-L1 on the surface of tumor cells, which gives the exosomal PD-L1 an opportunity to induce immune escape. For example, exosomes transport PD-L1 from PD-L1^+^ to PD-L1^−^ breast cancer cells and that exosomal PD-L1 binds to PD-1 on T cells to inhibit T cell activation (9). This may be one of the reasons for the resistance of PD-1/PD-L1 antibody. Anti-exosomal PD-L1 therapy may generate systemic anti-tumor immunity and may overcome resistance to current anti-PD-L1/PD-1 therapy in the future. The combination of suppression of exosome secretion and anti-PD-L1 antibody therapy may be more effective; thus, exosomal PD-L1 represents a promising therapeutic target in immunotherapy resistance.

Of course, resistance is not the only mechanism due to which immunotherapy fails. In some tumors, immune cells cannot reach the focus, no T cells are found in tumor parenchyma and stroma. Immunotherapy has no response to this “immune-desert tumor,” which is also a reason for immunotherapy resistance. There are many other unknown mechanisms to explore.

In view of the high potential and benefits of using exosomal PD-L1 for immunotherapy, exosomal PD-L1 has been registered for clinical trials. Phase I/II clinical trials related to exosomal PD-L1 have been recruiting cancer patients (Supplemental Table).

## Conclusion and Perspectives

In this review, we have systematically summarized and discussed the latest studies on exosomal PD-L1, including the biogenesis and transport of exosomal PD-L1, its role in immunosuppression, and the mechanism by which it inhibits T cells. We have also analyzed the potential of exosomal PD-L1 as a tumor biomarker for improving the efficacy of immunotherapy, and have highlighted the implications of using exosomal PD-L1 for immunotherapy resistance.

The expression level of PD-L1 in different tumor types is associated with poor prognosis, thus the expression level of PD-L1 should be an important predictor of therapeutic effect of the PD-1 pathway (71–74). As mentioned before, the CheckMate032 trial indicated that the expression of PD-L1 on tumor cells is not enough to judge the efficacy of immunotherapy (58). Moreover, PD-L1 expression may not be reliable due to problems with detection methods and tumor heterogeneity.

**Figure 4:**
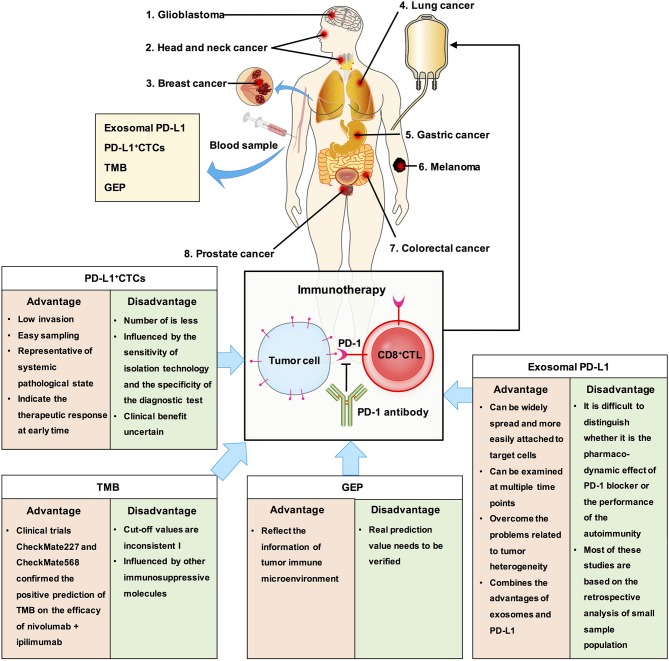
The clinical significance of exosomal PD-L1 and other biomarkers in cancer. We summaried the advantages and disadvantages of measurements as predictive biomarkers for immunotherapy. Exosomal PD-L1, which can serve as a tumor biomarker, combines the advantages of exosomes and PD-L1.

Other factors that can be detected using liquid biopsy and are also predictive biomarkers for immunotherapy include circulating tumor cells (CTCs), tumor mutational burden (TMB), gene expression profile, and exosomal PD-L1 (Figure 4). CTCs have the advantages of detection in liquid biopsy, low invasion, and easy sampling, and they are representatives of systemic pathological state. Measuring the dynamic changes in CTCs could indicate the effectiveness therapeutic response at an early time. PD-L1^+^ CTCs are promising candidates for determining the response to different therapeutic approaches. However, CTCs exist in peripheral blood, which has a complex environment, and the number of CTCs is less, usually 1–10/mL (75–77). The clinical significance of CTC analysis is strongly determined by the sensitivity of the CTC isolation technology and specificity of the diagnostic test to discriminate cells with malignant features. To this end, the clinical benefit of immune checkpoint blockade using PD-L1^+^ CTCs remains uncertain.

As early as 2015, TMB has been reported to be related to the efficacy of PD-1 inhibitors (78). TMB analysis showed that tumors with higher TMB, such as melanoma, NSCLC, head and neck squamous cell carcinoma, and bladder cancer, had higher response rates to immunotherapy, while tumors with lower TMB, such as pancreatic cancer and prostate cancer, had lower response rates to immunotherapy (79, 80). This phenomenon is related to neoantigens. The higher the TMB, the more likely it is for the tumor cells to express specific antigens for immune cell recognition and attack, and immunotherapy has a better therapeutic effect (81, 82). Recent studies have reported that TMB can be assessed using circulating tumor DNA and can be used as a potential marker of clinical benefit in patients with NSCLC receiving anti-PD-1 therapy (83). TMB can predict the overall survival rate of patients with multiple cancers. However, TMB did not solve all the problems. For example, the prediction of clinical benefits of TMB and immunotherapy cannot be accurately quantified; the value corresponding to high TMB varies among different cancers (84). Additionally, tumors with low TMB may enhance the immunogenicity of their neoantigens by epigenetic modification, while those with high TMB may have other immunosuppressive molecules in their immune microenvironment which may influence the efficacy of immunotherapy (1, 85, 86). Therefore, there may be some deficiencies in using TMB alone to predict the efficacy of immunotherapy. Moreover, there is same problem with using TMB as with using PD-L1, which is lack of clarity in selection of cut off value and in selection of patients who are not suitable for immunotherapy.

TMB is an indicator of tumor antigenicity, while PD-L1 and gene expression profile (GEP) reflect the status of tumor immune microenvironment. T cell-inflamed GEP has been proposed as a new biomarker to predict the efficacy of pembrolizumab in multiple solid tumors. It is composed of 18 inflammatory related genes, and similar to TMB, carries a lot of quantitative information (87). A study has reported the difference and connection between TMB and T cell-inflamed GEP in predicting the responders and non-responders to pembrolizumab by combining clinical cohort study and TCGA big data analysis, and finally confirmed that the best model for prediction of immune efficacy is combination of detection of TMB and T cell-inflamed GEP (88). However, its real prediction value needs to be verified later.

Although recent studies found that exosomal PD-L1 is related to the efficacy of PD-1 or the prognosis of the disease, there are still certain limitations. First, during PD-1 treatment, it is difficult to distinguish whether the effect is the pharmacodynamic effect of PD-1 blocker or performance of the autoimmunity. Second, most of these studies are based on the retrospective analysis of small sample population, and the results need to be verified by large-scale clinical prospective trials. Third, so far, no specific method has been developed to deal with the exosomal PD-L1 that is secreted by tumor cells.

Exosomal PD-L1 represents a major conceptual advancement, and it has interesting prospects in future research and clinical application. However, we do not thoroughly understand the mechanism by which exosomal PD-L1 inhibits immunity. Future studies should address the following urgent issues: (i) development of functional assays determining the effect of exosomal PD-L1 on other immune cells (e.g., NK cells, DCs, B cells, regulatory T cells, effector T cells, and macrophages). (ii) Understanding how exosomal PD-L1 affects tumor biological behavior through other mechanisms. Further studies are needed to identify the mechanism involved in induced exosomal PD-L1 expression and the consequent effects on cancer progression. (iii) We should investigate the autoimmunity against exosomal PD-L1 in patients and assess the ability of current methods to detect small amounts exosomal PD-L1 in plasma. Perhaps ongoing or future methods, such as digital droplet PCR (ddPCR), may overcome these issues.

There has been gradual integration of artificial intelligence and medicine; hence, perhaps with the help of advanced algorithms, we can solve the puzzle of immunotherapy, as just targeting driving genes is more like looking for a needle in the sea, which may end in nothing. The complexity of immune microenvironment seems to remind us that models that are more comprehensive should be used to evaluate immunotherapy rather than the old way of targeting driving genes to find a suitable biomarker. Future research will show whether exosomal PD-L1 together with other blood biomarkers can be used as blood-based biomarkers for selection of patients for immunotherapy.

## Author Contributions

YT wrote the manuscript and drew figures. PZ and YuW summarized the tables and revised the manuscript. JW, MS, YiW, LZ, JZ, WX, and ZZ helped to revise the manuscript. QL, SN, and YZ helped to designed and revise the manuscript. All the authors read and approved the final version of the review.

### Conflict of Interest

The authors declare that the research was conducted in the absence of any commercial or financial relationships that could be construed as a potential conflict of interest.
