# Supplementary material for: The Biogenesis, Biology, and Clinical Significance of Exosomal PD-L1 in Cancer
Source: Front Immunol. 2020 Apr 7;11:604. doi: 10.3389/fimmu.2020.00604 (PMC7158891; doi:10.3389/fimmu.2020.00604)
Supplement: Supplementary file 1 [file Table_1.docx]

**Table 1. Clinical trials of exosomal PD-L1 in cancer**

| **Patient Population** | **Phase** | **Status** | **Participants** | **Treatment** | **Outcome Measures** | **ClinicalTrials.gov Identifier** |
| --- | --- | --- | --- | --- | --- | --- |
| OSAS, Obstructive Cancer | NA | Not yet recrulting | 90 | Diagnostic test: Liquid biopsy | Exosomal PD1 / PD-L1 expression | NCT02890849 |
| NSCLC | I | Unknown | 60 | Liquid biopsy | Explore the consistency analysis of PD-L1 expression level detected in cancer tissues and exosome | NCT02890849 |
| NSCLC | I | Unknown | 60 | Five kinds of radiation-division with bioequivalent doses | The match rate of PD-L1 expression in tumor tissue and PD-L1 mRNA expression in plasma exosomes before / after radiotherapy | NCT02869685 |
| Lymphoma | I/II | Recrulting | 48 | Pembrolizumab given intravenously at a fixed dose of 200 mg every 3 weeks for up to 36 cycles | PD1/PD-L1 expression on TILs and exosomes | NCT02535247 |
| CRC | II | Recrulting | 40 | Participants received SBRT (BED>80Gy) to oligometastatic lesions and then receive Toripalimab (240mg)/q3w till progression of disease | Change of PD-L1 on exosomes/CTCs | NCT03927898 |

Abbreviations: OSAS, obstructive sleep apnea syndromes; NSCLC, non-small cell lung cancer; NA, not applicable; TILs, tumor infiltrating lymphocytes; CRC, colorectal cancer; SRBT, stereotactic body radiation therapy; CTCs, circulating tumor cells
